# Associations of sedentary behavior and physical activity with older adults’ physical function: an isotemporal substitution approach

**DOI:** 10.1186/s12877-017-0675-1

**Published:** 2017-12-06

**Authors:** Akitomo Yasunaga, Ai Shibata, Kaori Ishii, Mohammad Javad Koohsari, Shigeru Inoue, Takemi Sugiyama, Neville Owen, Koichiro Oka

**Affiliations:** 1 Faculty of Liberal Arts and Sciences, Bunka Gakuen University, 3-22-1 Yoyogi, Shibuya-ku, Tokyo, 151-8523 Japan; 2 Faculty Health and Sport Sciences, University of Tsukuba, Tsukuba, Japan; 3 Faculty of Sport Sciences, Waseda University, Tokorozawa, Japan; 4 Behavioural Epidemiology Laboratory, Baker Heart and Diabetes Institute, Melbourne, Australia; 5 Institute for Health & Aging, Australian Catholic University, Melbourne, Australia; 6 Department of Preventive Medicine and Public Health, Tokyo Medical University, Tokyo, Japan; 7 Swinburne University of Technology, Melbourne, Australia

**Keywords:** Accelerometer, Active behaviors, Functional test, Mobility, Sitting

## Abstract

**Backgrounds:**

The purpose of this study was to examine, in a sample of Japanese older adults, the associations of objectively-assessed sedentary behavior (SB) and physical activity (PA) with performance-based physical function. The isotemporal substitution (IS) approach was used to model simultaneously the effects of the specific activity being performed and the activity being displaced, in an equal time-exchange manner.

**Methods:**

Among 287 older adults (65–84 years), we used accelerometers to identify the daily average time spent on SB (≤1.5 METs); light-intensity PA (LIPA) (>1.5 to <3.0 METs); and moderate- to vigorous-intensity PA (MVPA) (≥3.0 METs). Physical function was assessed using five performance-based measures: hand grip strength, usual and maximum gait speeds, timed up and go, and one-legged stance with eyes open. We employed three linear regression models – a single-activity model, a partition model, and an IS model – to assess the associations of SB, LIPA, and MVPA with each of the five measures of physical function.

**Results:**

There were significant positive associations in the single-activity and partition models between MVPA and the measures of physical function (with the exception of hand grip strength). The IS models found that replacing SB or LIPA with MVPA was significantly and favorably associated with physical function measures.

**Conclusions:**

These findings indicate that replacing small amounts of SB and LIPA with MVPA (such as 10 min) may contribute to improvements in older adults’ physical function.

## Background

Physical function is a key component of health for older adults. Decline in physical function has been found to be closely related to disability and mortality [1, 2]. With the progression of aging in the world, extending healthy life expectancy, which is defined as the length of time an individual lives without limitations in their daily activities, is increasingly seen as a top priority in public health (e.g., Healthy People 2020 in the U.S.A., World Report on Aging and Health by WHO, and the National Health Promotion Movement in the twenty-first century in Japan) [3–5]. Therefore, more research to better understand factors that can help older adults maintain their physical function is needed to contribute to this priority [6].

Moderate- to vigorous-intensity physical activity (MVPA) is favorably associated with older adults’ physical function [7, 8]. Recent studies have shown that light-intensity physical activity (LIPA: e.g., some household work and slow walking) and sedentary behavior (SB: e.g., television viewing, computer use, and sitting in cars) are related to physical function in older adults [9–12]. For example, a longitudinal study examining associations of physical activity (PA) levels with functional status among older women found those who kept being active during a 14-year period had the best functional status [13]. Another study examining the cross-sectional associations of objectively-measured PA with physical health (including physical function) found LIPA to be positively associated with physical health among older adults [14]. Another cross-sectional study found older adults with lower levels of SB and a greater number of breaks in sitting time had significantly better physical function adjusting for MVPA [11].

However, there is a methodological concern in previous studies examining the relationships between PA levels and physical function. Since the total hours in a day are fixed; SB, LIPA, and MVPA are inter-related: increasing one behavior results in a reduction of other behaviors. Such substitutional relationships between behaviors need to be considered when research examines how a particular activity is associated with health outcomes. Nevertheless, there is only one previous study examining the potential benefits on older adults’ physical health when SB is replaced with LIPA or MVPA [9]. Buman et al. [9] found replacing 30 min/day of SB with LIPA to be positively associated with physical health, including physical function, among older adults. However, physical health in their study was assessed using a self-reported questionnaire, which has several limitations in terms of accuracy, validity, and reproducibility [15, 16]. There is thus the need to examine how performance-based physical function among older adults is associated with SB, LIPA and MVPA, considering their complementary relationships.

The aim of this study was to examine the associations of objectively-assessed SB, LIPA, and MVPA with performance-based physical function in a sample of Japanese older adults, using an isotemporal substitution (IS) approach to model simultaneously the effects of the specific activity being performed and the activity being displaced, in an equal time-exchange manner.

## Methods

### Participants and procedures

Participants were drawn from a larger epidemiological study conducted in Matsudo city, Chiba Prefecture, Japan. Matsudo city is located 20 km north of Tokyo with approximately half a million residents in 2016. To recruit the participants, first a randomly-selected 3000 people aged 65 to 84 years from the registry of residential addresses were contacted by an invitation letter. Of these, 951 agreed to participate in the main study, and 349 took part in a sub-study, in which PA and physical function were assessed objectively. Participants received a book voucher as compensation. All participants provided written informed consent. The study was approved by the Waseda University Institutional Committee on Human Research (2013–265) and the Institutional Review Board of Chiba Prefectural University of Health Sciences (2012–042).

### Physical activity and sedentary behavior

The participants’ PA was assessed using an accelerometer (Active style Pro HJA-350IT, Omron Healthcare, Kyoto, Japan). The detailed algorithm and validity of the accelerometer device have been described elsewhere [17–19]. Briefly, the device (74 × 34 × 46 mm; 60 g) measures anteroposterior (x-axis), mediolateral (y-axis), and vertical (z-axis) acceleration signals. The integral of the absolute value of the accelerometer output during a 60-s interval was calculated and converted into the total energy expenditure. The device estimates the intensity of activity by METs using a built-in algorithm. The CSV data files from the accelerometer were downloaded by Omron health management software BI-LINK for PA professional edition version 1.0 and then the files were processed by custom software (Custom-written Macro program for compiling data). A previous study, in which METs for household and locomotive activities were calculated, reported a linear relationship between filtered synthetic accelerations with PA intensity [18]. Participants were asked to wear the accelerometer on their waist for at least 7 days —except when sleeping or during water-based activities (e.g., bathing, showering, and swimming). To be eligible, participants needed to wear the accelerometer for ≥4 days (including 1 weekend day), with at least 10 h/day of wear time each day [20]. Non-wear time was defined as at least consecutive 60 min of 0 counts per minute (cpm), with allowance for up to 2 min of some limited movement (<50 cpm) within those periods [20]. For those who met the inclusion criteria, the daily average time spent on SB (≤1.5 METs), LIPA (>1.5 to <3.0 METs) and MVPA (≥3.0 METs) were calculated. These MET levels have been used by previous studies examining functional decline among older adults [21, 22].

### Physical function

Physical function was assessed using five performance-based functional tests: hand grip strength (upper body strength), usual and maximum gait speeds (gait speed), timed up and go (TUG) (mobility), and one-legged stance with eyes open (balance). Hand grip strength was measured using a Smedley-type handgrip dynamometer (TKK5041, Takei Scientific Instruments Co., Ltd., Niigata, Japan). We chose this type of handgrip device (rather than the Jamar dynamometer), because it is the most widely used method for evaluating hand grip strength in Japanese health studies [23]. Participants stood with their arms hanging naturally at their sides holding the dynamometer with the grip size adjusted to a comfortable level. They were instructed to squeeze the handgrip as hard as possible. Participants performed one trial with the dominant hand (to the nearest 0.1 kg). Usual and maximum gait speeds were measured using an 11 m course. Participants started walking at their normal or maximum paces, and time to walk 5 m was measured, starting when the body trunk passed the 3-m mark and ending when the body trunk passed the 8-m mark. Gait speed was calculated as distance divided by walking time (m/s). Usual gait speed was measured twice, and the faster of the two results (to the nearest 0.1 m/s) was used. Maximum gait speed was measured once. TUG measured the time for participants to complete the following sequence: start from a seated position in an armless chair; stand up from the chair and walk as fast as possible toward a pole placed 3 m in front of the chair, turn around the pole; and return to the chair and sit. TUG was conducted twice, and the faster of the two results (to the nearest 0.1 s) was used. One-legged stance with eyes open was measured using a participant’s preferred leg. Participants raise one leg and stand as long as possible. They were timed until they lost their balance or reached the maximum of 60 s. Participants performed two trials, and the longer of the two results (to the nearest 0.1 s) was used.

### Covariates

The following individual-level variables were considered as potential confounders: age, gender, body mass index (BMI), the number of past illnesses, complications and comorbidity, smoking status, drinking status, living arrangement, and highest educational attainment. The BMI was calculated using objectively-measured height and weight.

### Statistical analysis

Three multiple linear regression models including single-activity model, partition model, and IS model were used to examine the associations of SB, LIPA, and MVPA for each of the five items of physical function. We used 10 min as a unit for activity, thus the IS models examined the effect of replacing a 10-min of one activity with the same amount of another activity. This time unit was chosen for two reasons; this is the minimum amount of time in which activities should be accrued to meet current PA guidelines [7] and Japanese official PA guidelines for health promotion recommended trying to move for an additional 10 min a day for longer healthy life expectancy [24].

The single-activity model assessed each activity component separately (e.g., SB only), without taking into account the other activity types, but adjusting for total wear time and confounders. The model (in the case of SB) is expressed as follows:

Outcome variable = (b0) SB + (b3) total wear time + (b4) covariates.

The partition model examined all the behaviors simultaneously, without adjusting for total wear time. It is expressed as follows:

Outcome variable = (b0) SB + (b1) LIPA + (b2) MVPA + (b4) covariates.

In this model, the coefficient for one type of activity represents the effect of increasing this type of activity while holding the other activities constant. Since the total wear time is not included in the model (thus is not held constant), it represents the effects of adding rather than substituting an activity type.

The IS model estimates the effect of substituting one activity type with another for the same amount of time (e.g., replacing MVPA with SB, by removing SB from the model). The IS model (in the case of omitting SB from the model) is expressed as follows:

Outcome variable = (b1) LIPA + (b2) MVPA + (b3) total wear time + (b4) covariates.

The coefficients b1 and b2 in this model represent the effect of a 10-min substitution of SB with one of the activity types (LIPA or MVPA) while holding the other activity types and total wear time constant. For instance, b1 can be interpreted as the effect of replacing SB with LIPA for 10 min while holding MVPA and total wear time constant.

All the statistical contrasts were made at the 0.05 level of significant. Analyses were conducted using IBM SPSS Statistics 20.0 (IBM Japan Corp., Tokyo, Japan).

## Results

After excluding participants with missing covariates and lacking valid PA accelerometer data, the final sample consisted of 287 people (180 men, 107 women). Table 1 shows the characteristics of the study sample. The mean number of valid days of participant’s wearing accelerometer was 7.2 days (SD: 0.9). On average, participants wore accelerometer for 15 h/day, and the mean proportion of SB, LIPA, and MVPA duration to total accelerometer wear time were 58%, 36%, and 6%, respectively. These proportions were almost similar with those reported (the mean proportion of SB, LIPA, and MVPA duration were 64%, 31%, and 5% in men, and 54%, 41%, 5% in women) by the only previous study examined patterns of objective SB and PA among Japanese older adults [25]. Correlation coefficients were −0.67 between SB and LIPA, −0.36 between SB and MVPA, and 0.24 between LIPA and MVPA. Scores of performance-based functional tests (except for maximum gait speeds and TUG) were relatively high compared with the sample of Japanese community older population [26, 27].

**Table 1:** Participant Characteristics

	M (SD) or n, %	
Gender		
Men	180	62.7%
Women	107	37.3%
Age (years)	74.4	(5.2)
Body mass index (kg/m^2^)	23.5	(3.2)
Smoking		
Current smoker	21	7.3%
Never smoked or past smoker	266	92.7%
Drinking		
Current drinker	156	54.4%
Non-drinker, past drinker	131	45.6%
Residence status		
Living alone	33	11.5%
Living with someone	131	88.5%
Highest educational attainment		
University, junior college, vocational school, or higher level degree	110	38.3%
High school, or lower	177	61.7%
The number of past illnesses, complications and comorbidity	1.5	(0.9)
Activity		
SB (minutes/day)	522.7	(113.4)
LIPA (minutes/day)	328.7	(101.4)
MVPA (minutes/day)	50.2	(33.5)
Total wear time (minutes/day)	901.1	(87.5)
Physical function		
Hand grip strength (kg)	27.4	(8.3)
Usual gait speed (m/s)	1.3	(0.2)
Maximum gait speed (m/s)	1.8	(0.3)
Time up and go (second)	6.2	(1.2)
One-legged stance with eyes open (second)	42.9	(21.7)

*Note*. SB = Sedentary behavior, LIPA = Light-intensity physical activity, MVPA = Moderate- to vigorous- intensity physical activity

In the single-activity model (Table 2), MVPA was significantly and favorably associated with usual (*p* < .001) and maximum gait speeds (*p* < .001), TUG (*p* < .001), and one-legged stance with eyes open (*p* < .01). SB was significantly and negatively associated with usual (*p* < .01) and maximum gait speeds (*p* < .05) and TUG (*p* < .01). No significant association was observed for LIPA. In the partition model (Table 3), MVPA was the only activity that was associated with physical function outcomes. It was significantly and favorably associated with usual (*p* < .001) and maximum gait speeds (*p* < .001), TUG (*p* < .001), and one-legged stance with eyes open (*p* < .001). In the IS model (Table 4), each 10-min unit of SB or LIPA replaced with MVPA were significantly and favorably associated with usual (replacing SB: *p* < .001, replacing LIPA: *p* < .01) and maximum gait speeds (replacing SB: *p* < .001, replacing LIPA: *p* < .001), TUG (replacing SB: *p* < .001, replacing LIPA: *p* < .001), and one-legged stance with eyes open (replacing SB: *p* < .01, replacing LIPA: *p* < .01).

**Table 2:** Single-activity Models Examining the Associations of Each of SB, LIPA, and MVPA with Physical Function

	SB		LIPA		MVPA	
	*β*	95%CI	*β*	95%CI	*β*	95%CI
Hand grip strength	−0.056	(−0.130, 0.017)	0.058	(−0.024, 0.141)	0.092	(−0.135, 0.318)
Usual gait speed	−0.003	(−0.006, −0.001) **	0.001	(−0.001, 0.004)	0.019	(0.011, 0.026) ***
Maximum gait speed	−0.004	(−0.007, −0.001) *	0.002	(−0.002, 0.005)	0.025	(0.016, 0.034) ***
Time up and go	0.021	(0.008, 0.034) **	−0.011	(−0.025, 0.004)	−0.155	(−0.153, −0.077) ***
One-legged stance with eyes open	−0.238	(−0.477, 0.001)	0.139	(−0.131, 0.409)	1.187	(0.462, 1.913) **

*Note*. * *p* < .05, **p < .01, ***p < .001. SB = sedentary behavior, LIPA = light-intensity physical activity, MVPA = moderate- to vigorous-intensity physical activity. Regression coefficients correspond to a 10-min increment of each activity

Adjusted for age (years), sex, BMI (kg/m2), the number of past illnesses, complications and comorbidity, smoking status (current smoker/never smoked, past smoker), drinking status (current drinker/non-drinker, past drinker), residence status (living alone/living with someone), highest educational attainment (up to and including high school / university, junior college, vocational school, or higher degree), and total wear time

**Table 3:** Partition Models Examining the Associations of SB, LIPA, and MVPA with Physical Function

	SB		LIPA		MVPA	
	*β*	95%CI	*β*	95%CI	*β*	95%CI
Hand grip strength	0.033	(−0.052, 0.118)	0.088	(−0.012, 0.188)	0.099	(−0.140, 0.339)
Usual gait speed	0.000	(−0.003, 0.002)	0.000	(−0.003, 0.003)	0.018	(0.010, 0.026) ***
Maximum gait speed	0.000	(−0.003, 0.004)	0.000	(−0.004, 0.004)	0.025	(0.015, 0.035) ***
Time up and go	0.001	(−0.014, 0.015)	−0.003	(−0.019, 0.014)	−0.113	(−0.153, −0.072) ***
One-legged stance with eyes open	0.242	(−0.031, 0.514)	0.308	(−0.013, 0.629)	1.399	(0.630, 2.167) ***

*Note*. * *p* < .05, **p < .01, ***p < .001. SB = sedentary behavior, LIPA = light-intensity physical activity, MVPA = moderate- to vigorous-intensity physical activity. Regression coefficients correspond to a 10-min increment of each activity

Adjusted for age (years), sex, BMI (kg/m2), the number of past illnesses, complications and comorbidity, smoking status (current smoker/never smoked, past smoker), drinking status (current drinker/non-drinker, past drinker), residence status (living alone/living with someone), and highest educational attainment (up to and including high school / university, junior college, vocational school, or higher degree)

**Table 4:** Isotemporal Substitution Models Examining the Associations of Replacing SB, LIPA, and MVPA on Physical Function

	SB		LIPA		MVPA	
	*β*	95%CI	*β*	95%CI	*β*	95%CI
Hand grip strength						
Replace SB with	Dropped		0.054	(−0.030, 0.138)	0.066	(−0.164, 0.296)
Replace LIPA with	−0.054	(−0.139, 0.030)	Dropped		0.011	(−0.247, 0.270)
Usual gait speed						
Replace SB with	Dropped		0.000	(−0.003, 0.003)	0.018	(0.011, 0.026) ***
Replace LIPA with	0.000	(−0.003, 0.003)	Dropped		0.018	(0.010, 0.027) **
Maximum gait speed						
Replace SB with	Dropped		0.000	(−0.003, 0.004)	0.025	(0.016, 0.034) ***
Replace LIPA with	0.000	(−0.004, 0.003)	Dropped		0.025	(0.014, 0.035)***
Time up and go						
Replace SB with	Dropped		−0.003	(−0.018, 0.011)	−0.113	(−0.152, −0.074) ***
Replace LIPA with	0.003	(−0.011, 0.018)	Dropped		−0.110	(−0.154, −0.066) ***
One-legged stance with eyes open						
Replace SB with	Dropped		0.066	(−0.204, 0.355)	1.156	(0.418, 1.894) **
Replace LIPA with	−0.067	(−0.337, 0.203)	Dropped		1.088	(0.259, 1.917)**

*Note.* * *p* < .05, **p < .01, ***p < .001. SB = sedentary behavior, LIPA = light-intensity physical activity, MVPA = moderate- to vigorous-intensity physical activity. Regression coefficients correspond to a 10-min increment of each activity

Adjusted for age (years), sex, BMI (kg/m2), the number of past illnesses, complications and comorbidity, smoking status (current smoker/never smoked, past smoker), drinking status (current drinker/non-drinker, past drinker), residence status (living alone/living with someone), highest educational attainment (up to and including high school / university, junior college, vocational school, or higher degree), and total wear time

## Discussion

This study examined the associations of objectively-measured SB, LIPA, and MVPA with performance-based physical function measures among Japanese older adults. Several recent public health recommendations advocate reducing SB and increasing MVPA for maintaining better health [24, 28]. Our analysis using the IS model provides empirical evidence supporting these recommendations.

Previous studies have consistently reported that MVPA has favorable effects on various health outcomes including physical function in older adults [7, 29]. Consistent with these studies, we found MVPA to be positively and significantly associated with older adults’ physical function (except for hand grip strength) in a single-activity model (total time held constant) and a partition model (other activity variables held constant). In addition, our findings showed that replacing SB or LIPA with MVPA had beneficial effects on participants’ physical function, except for hand grip strength. These results indicated that replacing a small amount of SB or LIPA (such as 10 min) with equal time of MVPA during one day was associated with 1.4 to 2.7% better function scores. It should be noted that no significant associations were observed for hand grip strength, which is often used as an overall measure of body strength [23]. This is plausible as PA measured in this study mainly involves lower limb muscles, for which mixed associations with hand grip strength have been reported [30]. It has been also found that correlation between hand grip strength and lower limb muscle strength tends to be weaker among older adults compared with younger adults [31]. Several previous studies using the IS approach reported benefits of replacing relatively long time of SB (e.g. 30 to 60 min) with MVPA on health outcomes [32, 33]. However, replacing 30 or 60 min of SB with MVPA during one day may be practically difficult for older adults.

Recent studies have demonstrated the favorable effects of LIPA on health in older population [9, 12]. Based on these findings, it is possible to posit that replacing SB with LIPA may be beneficial to older adults’ physical function. However, our results did not show beneficial effects of replacing SB with LIPA. In addition, the regression coefficients in the IS models where SB was replaced with MVPA and that where LIPA was replaced with MVPA were almost the same for gait speeds and TUG. Several studies found MVPA to have beneficial effects on muscle strength, but not LIPA [34, 35]. Better physical function requires sufficient muscle strength, which may not be obtained by LIPA. In addition, the LIPA in our study may include household chores (e.g., cooking, washing dishes, ironing, and house cleaning). It is possible that the nature of these activities may have contributed to lack of a significant improvement in physical function when they are substituted by sitting time. This suggests that LIPA may be associated with self-reported or mental health of older adults, but may not be intense enough to be related to their physical function. Another possibility is misclassification of activity intensity in previous studies: LIPA may have included relatively strong intensity activities such as gardening [36]. Also, some previous studies classified objectively-measured LIPA into two categories: low-light intensity PA (>1.5 to <2.0 METs) and high-light intensity PA (≥2.0 to <3.0 METs), and reported that they are associated differently with health outcomes including physical function [9, 14]. Such co-existence of activities with different intensities within LIPA may have played a role in the relationships observed in the study. Further research using comprehensive health outcomes is needed to understand whether and to what extent LIPA is beneficial to older adults’ health.

This study has some limitations. First, the accelerometer did not assess activities involving only upper or lower limbs without trunk movement (e.g., some household work and cycling) and water-based PA (e.g. swimming or underwater walking). Second, as a cross-sectional study, we are unable to infer a cause-and-effect relationship between SB, PA, and physical function. This is an important issue because behavior pattern may be a reflection of functional status. Future longitudinal studies are needed to address this limitation. Third, the participants in this study were not a representative sample of Japanese older (i.e., more active and healthy). Further research using an intervention design with a large and diverse sample is needed to further understand about cause-and-effect relationship between different levels of activity and older adults’ physical function. The strength of this study was the use of objectively-measured behaviors and performance-based physical function.

## Conclusions

Our findings indicated that replacing even small amounts of SB (e.g. watching TV and working at a desk) with MVPA (e.g. brisk walking and exercise/sport) may contribute to improvements in physical function in older adults. Potential favorable effects were identified for replacing only 10 min per day of SB with MVPA.

## Abbreviations

BMI: Body mass index
IS: Isotemporal substitution
LIPA: Light-intensity physical activity
MVPA: Moderate- to vigorous-intensity physical activity
PA: Physical activity
SB: Sedentary behavior
TUG: Timed up and go
